# Madelung’s Disease as an Example of a Metabolic Disease Associated with Alcohol Abuse—Diagnostic Importance of Computed Tomography

**DOI:** 10.3390/ijerph19095168

**Published:** 2022-04-24

**Authors:** Przemysław Jaźwiec, Maria Pawłowska, Karolina Czerwińska, Małgorzata Poręba, Paweł Gać, Rafał Poręba

**Affiliations:** 1 Specialist Medical Center in Polanica-Zdrój, Jana Pawła II 2, 57-320 Polanica Zdrój, Poland; przemkolog@wp.pl (P.J.); maria.pawlowska@yahoo.pl (M.P.); 2 Division of Environmental Health and Occupational Medicine, Department of Population Health, Wroclaw Medical University, Mikulicza-Radeckiego 7, 50-368 Wroclaw, Poland; karolina.czerwinska@student.umw.edu.pl; 3 Department of Paralympic Sports, Wroclaw University of Health and Sport Sciences, Witelona 25a, 51-617 Wroclaw, Poland; poreba1@wp.pl; 4 Department of Internal Medicine, Occupational Diseases and Hypertension, Wroclaw Medical University, Borowska 213, 50-556 Wroclaw, Poland; rafal.poreba@umw.edu.pl

**Keywords:** Madelung’s disease, lipomatosis, alcohol abuse

## Abstract

Madelung’s disease is a rare metabolic disorder characterized by a symmetrical accumulation of nonencapsulated adipose tissue deposits, mainly around the head, neck and shoulders. Fat deposits can grow and put pressure on other organs causing a variety of symptoms, inter alia, dysphagia, breathing difficulties, neck stiffness and headache. Madelung’s disease is often accompanied by other disorders such as diabetes, hypertension, hypothyroidism, or liver disease. In addition to somatic issues, mental health problems may also develop causing social exclusion and depression. Middle-aged men with a history of alcohol abuse are the most commonly affected. Various imaging techniques, including computed tomography (CT), are helpful in stating the diagnosis. This paper presents a case of a 33-year-old man with extensive adipose tissue overgrowth around neck and chest. CT-enhanced scans with multiplanar reconstruction (MPR) and volume rendering technique (VRT) reconstruction are also included.

## 1. Introduction

Madelung’s disease, also known as Benign Symmetrical Lipomatosis (BSL) or Launois–Bensaude syndrome, is a rare metabolic disorder. It is manifested by a symmetrical accumulation of nonencapsulated adipose tissue deposits, mainly around the head, neck and shoulders [1].

Fat deposits can grow rapidly (within months) or slowly over the years, causing a variety of symptoms, inter alia, dysphagia, breathing difficulties, neck stiffness and headache. BSL can occur in all ethnic groups but is usually found in Mediterranean and European populations. Men aged 30–60 years, chronically abusing alcohol are the most commonly affected [2].

The etiology of BSL is not fully known; however, an association with a defect in noradrenergic mitochondrial regulation of brown adipose tissue (BAT) is suggested. Differential diagnosis should primarily include obesity, goiter, Cushing’s disease and neoplastic changes. Surgical removal of lipomas or liposuction are the leading treatment methods; however, fat deposits tend to recur despite treatment [3].

The paper presents the case of Madelung’s disease as an example of a metabolic disease associated with alcohol abuse in order to emphasize the diagnostic importance of computed tomography in the assessment of morphology and the differentiation of this pathology.

## 2. Case Report

A 33-year-old obese man with arterial hypertension and alcohol dependence syn-drome (for over 10 years) presented to the Diagnostic Imaging Department for a neck and chest CT scan due to adipose tissue overgrowth.

The patient had a history of numerous enlarging subcutaneous tissue deposits growing in different areas of the body for about 5 years. Moreover, due to chronic chest pain, he often presented to the Emergency Department.

In March 2021, he was admitted to the Emergency Department for nonspecific abdominal pain after 11 days of excessive alcohol consumption. On admission, he was intoxicated (3.77 g/L), confused and had slurred speech, and his medical history was difficult to ascertain. He claimed to have had breathing problems for a long time, which he treated with alcohol. He was taking alprazolam (Afobam) chronically and underwent an alcohol abuse treatment. Physical examination revealed significant enlargement of the neck circumference (which was also reported during previous visits to the Emergency Department). The patient was circulatory and respiratory efficient, his abdomen was soft, nontender, and there was an excess of fatty tissue in the lower abdomen. His laboratory results were as follows: red blood cells (RBC) 3.70 million/µL [4.54–5.78]—indicating mild macrocytic anemia—hemoglobin (HGB) 12.3 g/dL [13.3–17.2], mean corpuscular volume (MCV) 98.1 fL [81.2–94], non-elevated inflammatory markers, significantly elevated levels of GGTP 2313 U/L [<55], AST 222 U/L [<35], ALAT 102 U/L [<50], and lipase 81 U/L [<67]. No prohibited substances were found in the drug panel that was performed. Abdominal ultrasound examination revealed fatty liver and gallbladder sludge. He was discharged from the department and advised to continue treatment on an outpatient basis.

In December 2021, he was consulted at the surgical clinic and endocrinology clinic. The examination confirmed adipose tissue overgrowth in many locations, i.e., within neck, shoulders, chest, lower abdomen. There was no evident muscle atrophy. Neck ultrasound showed numerous adipose tissue deposits and an inflamed lymph node (15 × 10 mm) around right mandibular angle with increased hilar vascularity. A CT scan of the neck and chest was ordered to assess the extent of the adipose tissue overgrowth.

**Figure 1:**
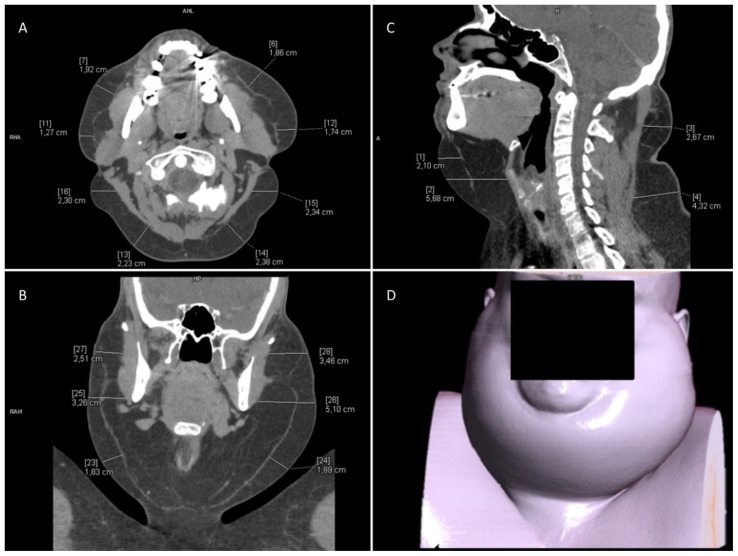
Computed tomography of the neck. (**A**) Axial reconstruction. (**B**) MPR reconstruction, frontal view. (**C**) MPR reconstruction, sagittal view. (**D**) VRT reconstruction.

CT-enhanced scans were obtained, following which multiplanar reconstruction (MPR) and volume rendering technique (VRT) reconstruction were created (see Figure 1).

Location of the fatty deposits and their thickness, based on the CT scan, are presented in Table 1 and Table 2. The values presented in the tables refer to the maximum thickness of adipose tissue measured in the indicated locations, with approximation to 5 mm.

Other CT findings included parotid and submandibular glands enlargement (with no noticeable focal changes), loss of physiological cervical lordosis, glandular breast tissue overgrowth, thoracic spine arthritis and Schmorl nodules. In general, the CT scan showed commonly reported imaging features of Madelung disease.

The patient was qualified for conservative treatment. Absolute prohibition of alcohol consumption (addiction treatment) and weight reduction were recommended. Moreover, the patient was referred for rehabilitation to improve physical efficiency.

## 3. Discussion

Madelung’s disease is also known as Benign Symmetrical Lipomatosis (BSL), Multiple Symmetrical Lipomatosis (MSL) or Launois–Bensaude syndrome [1,2]. It was first described by Benjamin Brodie in 1846. Over 40 years later, Otto Madelung (1888), and after another 10 years Pierre-Émile Launois and Raoul Bensaude (1898), presented more cases of patients with this condition [3]. 

Madelung’s disease is a rare disorder (1:25,000) characterized by an excessive symmetrical growth of unencapsulated fatty deposits around the head, neck, upper chest, arms, as well as hips and thighs [2,3]. The etiology of BSL is not fully understood [4]. Most often it affects men (M:F—15:1) aged 30–60, chronically abusing alcohol [5]. However, it has also been reported in women and non-alcohol users [6,7]. Cytopathy of brown adipose tissue and disturbances in lipid metabolism are considered as possible causes. It is suggested that excessive alcohol consumption may impair adrenergic lipolysis and lead to an uncontrolled deposition of fat masses in various parts of the body [1,2].

There are two main systems of classifying Madelung disease. The first one, created in 1984 by G. Enzi, distinguishes two types of fat masses distribution. Type 1, characterized by the location of changes in the nape, neck and shoulders, creating an image of the so-called “Horse collar” (“Madelung’s collar”). As a result, patients have a “pseudoathletic” appearance. Type 2, characterized by the location of fat masses in the abdomen, hips and thighs. The second classifying system, created in 1991 by G. Donhauser, divides BSL patients into four groups: type I (horsecollar), II (pseudo athletic type), III (gynecoid type), IV (abdominal type) [3].

Initially, aesthetic reasons encourage patients to seek medical advice. However, as the disease progresses, growing fat masses may put pressure on other structures, i.e., trachea, larynx, pharynx, esophagus or blood vessels, and cause symptoms. Depending on the severity of the disease, patients may experience breathing difficulties, speaking difficulties, dysphagia, as well as reduced neck mobility or general mobility [2]. Madelung’s disease is often accompanied by other disorders such as diabetes, hypertension, hypothyroidism, or liver disease. Neurological disorders in the form of polyneuropathy may also develop. They are the result of alcoholic demyelination and degeneration of axonal nerve fibers [4,5]. It should be remembered that in addition to somatic issues, BSL also affects mental health and can lead to social exclusion and depression.

The diagnosis of Madelung’s disease is symptomatic. Diagnosis involves the correlation of clinical data and images from diagnostic imaging. It is important to exclude other diseases with excessive adipose tissue. In response to the variety of possible symptoms differential diagnosis should consider numerous diseases, i.e., obesity, thyroid goiter, lipoma, liposarcoma, Cushing’s disease, cysts of the neck, diseases of the salivary glands, thyroid cancer, leukemia or soft tissue sarcoma [8]. BSL is characterized by a symmetrical distribution of adipose tissue, sparing the peripheral parts of the limbs, and by a history of excessive alcohol consumption. Ultrasound, computed tomography and magnetic resonance imaging are used to describe the location and extent of fatty deposits. A very important feature of BSL, which is confirmed in diagnostic imaging studies, is the lack of pathology boundaries from the surrounding tissues, e.g., no encapsulation. A biopsy is not required to confirm the diagnosis. However, it may be useful to rule out liposarcoma, for lesions characterized by a mixed nature—focal lesions among diffuse pathology [8].

BSL treatment can be divided into surgical and non-surgical methods. Surgical removal of fatty deposits is the most used treatment. The effects of surgery are often unsatisfactory and do not give long-term results. Patients may require multiple resections as the changes tend to recur. In some BSL cases, extensive lipectomy combined with liposuction or isolated liposuction is recommended [2,9]. 

Intralipotherapy, i.e., injection lipolysis, is one of the recommended methods of non-invasive treatment [9]. In this method phosphatidylcholine/sodium deoxycholate is applied directly to the adipose tissue [10]. 

Dietary treatment and manual therapy are usually ineffective. Weight loss can only limit the progression of the disease in some cases [4]. Limiting alcohol consumption does not reduce lipomas; nevertheless, it is recommended for its positive effect on the accompanying metabolic disorders and reduced mortality [4].

The presented case is an example of rare pathologies related to adipose tissue, which should be kept in mind in times of increasing obesity epidemic. At the same time, it is an example of an environmental and behavioral disease, an example indicating a less known mechanism of alcohol toxicity—a mechanism related to the influence of ethanol on the metabolism of adipose tissue. It can be an additional useful argument for the prevention of alcohol dependence. What is no less important is that the case shows the importance of modern methods of imaging diagnostics (in the discussed case computed tomography) in the diagnosis and differential diagnosis of the discussed pathology. Previously published articles mainly presented clinical data, often supplemented with photos from a physical examination. In the present article, we present high-quality computed tomography images, at the same time indicating the characteristics of the pathology that enable us, in correlation with clinical data, to make a diagnosis by excluding other diseases. The applied diagnostic procedure with the use of computed tomography also allows avoiding invasive diagnostics in most cases, which is also indicated by the presented case.

## 4. Conclusions

Computed tomography with MPR and VRT reconstructions is an important diagnostic tool for Madelung’s disease, enabling the assessment of the extent of lesions and treatment planning, as well as, in correlation with clinical data, differentiation with other diseases with excessive development of adipose tissue.

## Figures and Tables

**Table 1:** Computed tomography of the neck. The exact location of adipose tissue overgrowth and measurement of its thickness.

Neck CT	
Exact Location	Fat Layer Thickness [mm]
left side of the neck	50
right side of the neck	50
chin area	70
infrahyoid region	60
right neck triangle	40
left neck triangle	30

**Table 2:** Computed tomography of the chest. The exact location of adipose tissue overgrowth and measurement of its thickness.

**Chest CT—Anterior Part of the Chest**			
Right side		Left side	
Exact location	Fat layer thickness [mm]	Exact location	Fat layer thickness [mm]
Upper part	30	Upper part	20
Middle part	25	Middle part	15
Lower part	20	Lower part	15
**Chest CT—Lateral Part of the Chest**			
Right side		Left side	
Exact location	Fat layer thickness [mm]	Exact location	Fat layer thickness [mm]
Upper part	40	Upper part	30
Middle part	30	Middle part	40
Lower part	28	Lower part	30
**Chest CT—Posterior Part of the Chest**			
Right side		Left side	
Exact location	Fat layer thickness [mm]	Exact location	Fat layer thickness [mm]
Upper part	20	Upper part	20
Middle part	25	Middle part	25
Lower part	30	Lower part	35

## Data Availability

Not applicable.
